# Pneumococcal density and respiratory co-detection in severe pediatric pneumonia in Laos

**DOI:** 10.1038/s41598-025-01659-y

**Published:** 2025-05-21

**Authors:** Audrey Dubot-Pérès, Sue J. Lee, David A. B. Dance, Catherine Satzke, Kerryn Moore, Casey L. Pell, Belinda D. Ortika, Monica L. Nation, Eileen M. Dunne, Keoudomphone Vilivong, Toukta Bounkhoun, Souphatsone Phommachan, Ruth Lim, Jana Lai, Melinda Morpeth, Mayfong Mayxay, Xavier de Lamballerie, Paul N. Newton, Fiona M. Russell

**Affiliations:** 1 Unité des Virus Émergents (UVE: Aix-Marseille Univ, Università di Corsica, IRD 190, Inserm 1207, IRBA), Marseille, France; 2 Microbiology Laboratory, Lao-Oxford-Mahosot Hospital-Wellcome Trust Research Unit (LOMWRU), Mahosot Hospital, Vientiane, Lao PDR; 3 Centre for Tropical Medicine and Global Health, Nuffield Department of Clinical Medicine, University of Oxford, Oxford, UK; 4 Mahidol-Oxford Tropical Medicine Research Unit (MORU), Mahidol University Faculty of Tropical Medicine, Bangkok, Thailand; 5 Department of Infectious Diseases, The Alfred Hospital and School of Translational Medicine, Monash University, Melbourne, Australia; 6 Faculty of Infectious and Tropical Diseases, London School of Hygiene and Tropical Medicine, London, UK; 7 Department of Paediatrics, The University of Melbourne, Melbourne, VIC Australia; 8 Infection, Immunity and Global Health, Murdoch Children’s Research Institute, Melbourne, VIC Australia; 9 Department of Microbiology and Immunology, The University of Melbourne at the Peter Doherty Institute for Infection and Immunity, Melbourne, VIC Australia; 10 National Centre for Epidemiology & Population Health, Australian National University, Canberra, Australia; 11 The Royal Children’s Hospital, Melbourne, Australia; 12 Institute of Research and Education Development, University of Health Sciences, Vientiane, Lao PDR; 13 Saw Swee Hock School of Public Health, National University of Singapore, Singapore, Singapore

**Keywords:** Acute respiratory infection, Laos, Co-detection, Respiratory syncytial virus, Pneumococcal density, Bacteriology, Viral epidemiology, Infectious diseases, Respiratory tract diseases

## Abstract

There is growing evidence on the importance of bacterial/viral interaction in the course of pneumonia. In Laos, no study has investigated respiratory pathogen co-detection. We conducted a study at Mahosot Hospital in Vientiane to determine whether bacterial/viral co-detection and pneumococcal density are associated with severe pneumonia. Between December 2013 and December 2016, 934 under 5 years old hospitalized children with ARI were enrolled. Swabs from the upper respiratory tract were collected and analyzed by real-time PCR. The most common co-detected microorganisms were *Streptococcus pneumoniae*/*Haemophilus influenzae* (24%), Respiratory Syncytial Virus (RSV)/*S. pneumoniae* (12%) and RSV/*H. influenzae* (16%). Pneumococcal density was 4.52 times higher in influenza virus positive participants. RSV/*S. pneumoniae* and RSV/*H. influenzae* co-detections were positively associated with severe pneumonia in univariate analysis (OR 1.86, 95%CI:1.22–2.81, *p* = 0.003 and OR 2.09, 95%CI:1.46-3.00), but not confirmed in adjusted analysis (aOR 0.72, 95%CI:0.38–1.6, *p* = 0.309 and aOR 1.37, 95%CI:0.73–2.58). In RSV positive patients, there was no association between pneumococcal density and severe pneumonia. Our findings confirmed an association between pneumococcal density and influenza but not RSV severe pneumonia in young children. Results highlight the complexity of the interaction of viral/bacterial pathogens, which might not have a simple synergistic action in the evolution of pneumonia.

**Supplementary Information:**

The online version contains supplementary material available at 10.1038/s41598-025-01659-y.

## Introduction

Worldwide, acute respiratory infection (ARI) is the leading cause of morbidity and mortality in children aged one month to less than 9 years old^1^. Perin and colleagues estimated that lower respiratory infections caused 0.74 million deaths (14% of all deaths) in children less than 5 years old in 2019^1^. Despite the importance of ARI, understanding the aetiology is complex: this has yet to be fully characterized in high income countries and remains largely unknown in low-and middle-income countries (LMICs).

A wide diversity of bacterial and viral microorganisms have been identified as pathogens, either individually or in combination, causing respiratory tract illness. A recent systematic review on aetiologies of community acquired pneumonia in LMICs found that most pneumonia cases were due to respiratory syncytial virus (RSV), human metapneumovirus (HMPV), influenza, parainfluenza, *Streptococcus pneumoniae*, *Haemophilus influenzae*, *Staphylococcus aureus*, *Mycoplasma pneumoniae* and *Mycobacterium tuberculosis*. Bacteria (*S. pneumoniae*,* S. aureus*) were more often detected in severe than in non-severe cases of pneumonia. RSV was the leading viral cause of severe disease^2^.

Higher pneumococcal colonisation density in the upper respiratory tract (URT) facilitates the spread of pneumococci to the lung. Several studies conducted in children have shown that higher pneumococcal nasopharyngeal colonisation density was associated with disease, ARI, pneumonia or severe pneumonia^3–9^. Studies have shown that introduction of pneumococcal conjugate vaccines (PCV) led to an approximately 50% reduction of community-acquired alveolar pneumonia (CAAP) in young children, showing the important causative role of *S. pneumoniae* in CAAP. Therefore, with the introduction of the *Haemophilus influenzae* type b (Hib) vaccine and PCV and improvements in living standards, it is important to understand any changes in pneumonia aetiology over time.

Alteration of normal seasonal respiratory virus patterns during the COVID-19 pandemic shed some light on the role of viruses in respiratory infections. Studies from Israel and France found that during the COVID-19 pandemic, concomitantly to the full suppression or substantial decreases of RSV, influenza viruses, and HMPV infections, there was a decline in pneumococcal-associated diseases and that this was not predominantly related to reduced pneumococcal carriage and density^10–12^. This suggests that although pneumococcal carriage is a prerequisite in the development of pneumococcus-associated lung diseases, in many cases this is not sufficient. Viral co-infection also plays a key role in disease development.

Secondary bacterial infection following viral infection is well-documented and has been associated with more severe ARI presentation, such as pneumococcal pneumonia which has frequently been associated with influenza virus and RSV infections^13,14^. Viral infection is thought to have a role in the enhancement of the growth of *S. pneumoniae* colonizing the URT, leading subsequently to bacterial superinfection of the lower respiratory tract (LRT)^6,15,16^. A recent systemaic review describes the interactions between RSV and *S. pneumoniae* and their synergic role in the development of the disease in children^17^.

Between 2011 and 2014 the PERCH (Pneumonia Etiology Research for Child Health) case-control study was conducted in seven countries in Africa and Asia and enrolled 4232 hospitalized children (1 to 59 months old) with severe or very severe pneumonia^18^. Viruses were estimated to be responsible for 61.4% of the cases and bacteria for 27.3%. The mean number of different microorganisms detected by PCR in the same URT sample was 3.8 and all viral pathogens were co-detected with bacteria^19^.

In the Lao People’s Democratic Republic (PDR) (Laos), there is little information available on the burden or etiology of respiratory infection, particularly in children. Vaccination with the 13-valent pneumococcal conjugate vaccine (PCV13) was initiated in November 2013. A few studies have been conducted in the past two decades, mainly on viral infections, showing the importance of influenza virus and RSV infections^20–24^. Previously, we found that among children < 5 years old and hospitalized with ARI, severe pneumonia was positively associated with higher pneumococcal density among pneumococcal carriers^5^. However, none of these studies investigated respiratory pathogen co-detection. We conducted ARI surveillance at Mahosot Hospital, Vientiane identifying microorganisms detected in the upper respiratory tract of children presenting with ARI over three years (December 2013 to December 2016). In this analysis we aim to determine whether (i) co-detections (*S. pneumoniae*/RSV or *S. pneumoniae*/influenza virus) are associated with higher pneumococcal colonization (ii) co-detections (*S. pneumoniae*/*H. influenzae*, or *S. pneumoniae*/RSV or RSV/*H. influenzae*) are associated with severe pneumonia; (iii) in RSV positive patients, higher pneumococcal colonization density is associated with severe pneumonia.

## Methods

### Study site and patient recruitment

This prospective observational study was conducted from December 2013 to December 2016 at Mahosot Hospital, Vientiane, Laos, a 400-bed hospital providing primary, secondary, and tertiary care and admitting on average 2,000 patients each month.

Children aged 2 to 59 months old, admitted to a paediatric ward (general paediatric, paediatric infectious diseases, or paediatric intensive care unit (ICU)) were included in the study if they presented with: onset of symptoms less than 14 days, fever (axillary temperature > 38.0 °C) or history of fever, and at least one respiratory symptom (dyspnea, cough, rhinitis) or abnormal pulmonary auscultation on physical examination. Demographic, medical history, clinical and environmental data were collected using a questionnaire by physicians from our research team, by interviews, physical examination and consulting medical charts as described elsewhere^25^. Patients and their carers were interviewed within 48 h after hospital admission.

Pneumonia was defined as children with cough or difficulty breathing and fast breathing (aged 2–11 months: ≥50 breaths/minute, aged 1–4 years: ≥40 breaths/minute) or chest indrawing^26^. Severe pneumonia was defined as children with cough or difficulty breathing who had at least one of the following criteria: oxygen saturation < 90% while breathing room air, or central cyanosis; severe respiratory distress; signs of pneumonia with a general danger sign (inability to breastfeed or drink, lethargy or reduced level of consciousness, convulsions, vomiting)^26^.

### Ethics approval and consent to participate

We obtained written informed consent from the legal guardians of all patients before recruitment to the study. The study was conducted according to the protocol approved by the National Ethics Committee for Health Research, Ministry of Health, Vientiane, Laos, and the Oxford University Tropical Ethics Research Committee (Oxford, UK). The study has been performed in accordance with the Declaration of Helsinki.

### Sample collection

Nasopharyngeal, nasal and throat swab specimens were collected from all patients at the time of inclusion in the study (within 48 h after hospital admission). Swabs were placed separately in 1 mL skim milk, tryptone, glucose, and glycerol (STGG) medium (nasophyarngeal swabs) or viral transport medium (Sigma Virocult^®^, MWE, nasal and throat swabs)^27,28^. Samples were transported to the laboratory within 2 h of collection in a cool box. Swabs were squeezed, the media was mixed by pipetting and aliquoted and stored at − 80 °C before performing the laboratory assays.

### *S. pneumoniae* detection and quantification

Nasopharyngeal samples were shipped on dry ice to the Murdoch Children’s Research Institute, Melbourne, Australia for testing. DNA was extracted from 100 µL of STGG medium (following enzymatic treatment) using a MagNA Pure LC machine (Roche) using the DNA Isolation Kit III (bacteria, fungi) (Roche) as previously described^29^. Pneumococci were detected by probe-based real-time polymerase chain reaction (qPCR) targeting the *lytA* gene^29,30^. Samples with Ct > 40.0 were considered negative for pneumococcus detection; for all remaining samples, pneumococcal detection was confirmed by culture and microarray as described previously^29^. Following *lytA* qPCR, the bacterial density (reported as genome equivalents [ge]/mL) was estimated by reference to a standard curve of pneumococcal DNA with the assumption that each pneumococcal cell contains one 2-Mb genome, and each genome contains a single copy of the *lytA* gene^29,30^.

### Other respiratory microorganisms detection

100 µL of nasal swab and 100 µL of throat swab media were pooled together for each patient and extracted using the Cador Pathogen 96 QIAcube HT kit (Qiagen) following the manufacturer’s instructions, with an elution of 90 µL.

In the first year of the study (December 2013 to December 2014), extracts were tested for 33 pathogens using the FTD^®^ respiratory pathogens 33 kit (Fast-track Diagnostics) which consists of multiplexed reverse transcription qPCR (q(RT-)PCR) including, in addition to *S. pneumoniae* detection (see above), the detection of: Influenza A, B and C viruses; RSV A and B; *H. influenzae;* and *H. influenzae* type b.

To focus on the main pathogens of interest, from January 2015 to December 2016, in addition to *S. pneumoniae* detection (see above), extracts were tested using previously published singleplex q(RT)-PCR assays targeting 7 respiratory microorganisms including influenza A virus^31^, influenza B virus^32^, RSVA/B^33^, and *H. influenzae*^34^. For each system, primers and probe mix were lyophilized as single test format in plates as previously described^35^. Testing was performed using the iTaq™ Universal Probes One-Step reverse transcriptase kit (Bio-Rad), from 10 µL of nucleic acids, in a final reaction volume of 30 µL. The thermal cycling was: 10 min at 50 °C, 5 min at 95 °C, followed by 44 cycles of 15 s at 95 °C and 30 s at 60 °C.

Comparison of FTD^®^ respiratory pathogens 33 kit and RSV singleplex q(RT)-PCR assays using URT samples from 260 pediatric hospitalized patient showed 96% agreement (unpublished data).

All amplification and detection were performed with the CFX Real-time PCR system instrument (Bio-Rad). Positive and negative (no template) controls were included in each PCR run. The q(RT-)PCR assays were considered as positive if the Cq value was < 35.

### Statistical analysis

Data were double entered into an Access database (Microsoft Corporation). Following cleaning, statistical analysis was performed using Stata/SE version 14.0 (StataCorp, https://www.stata.com). Data were summarised using frequencies with percentages and medians with interquartile range (IQR). For unadjusted comparisons, the χ2 or Fisher’s exact test were used for categorical data and the Mann-Whitney U test for continuous variables, such as pneumococcal density.

Potential confounders were identified a priori using directed acyclic graphs (DAGs) (Supplementary Figs. S1, S2). Logistic regression models were used to quantify associations between co-detection (RSV/*S. pneumoniae*, or RSV/*H. influenzae*, or *H. influenzae*/*S. pneumoniae*) and severe pneumonia. In addition to factors identified in the DAGs, variables that were correlated with the outcome in the univariate analysis at *p* < 0.20 were included in adjusted logistic regression models. For models using *S. pneumoniae* density as outcome (i.e., associations with viral detection (RSV or influenza virus) and, in RSV positive patients, associations with severe pneumonia), negative binomial regression models were used. Results were reported as odds ratio (OR) and rate ratios (RR), with 95% confidence intervals.

## Results

### Characteristics of children hospitalized with ARI

**Fig. 1:**
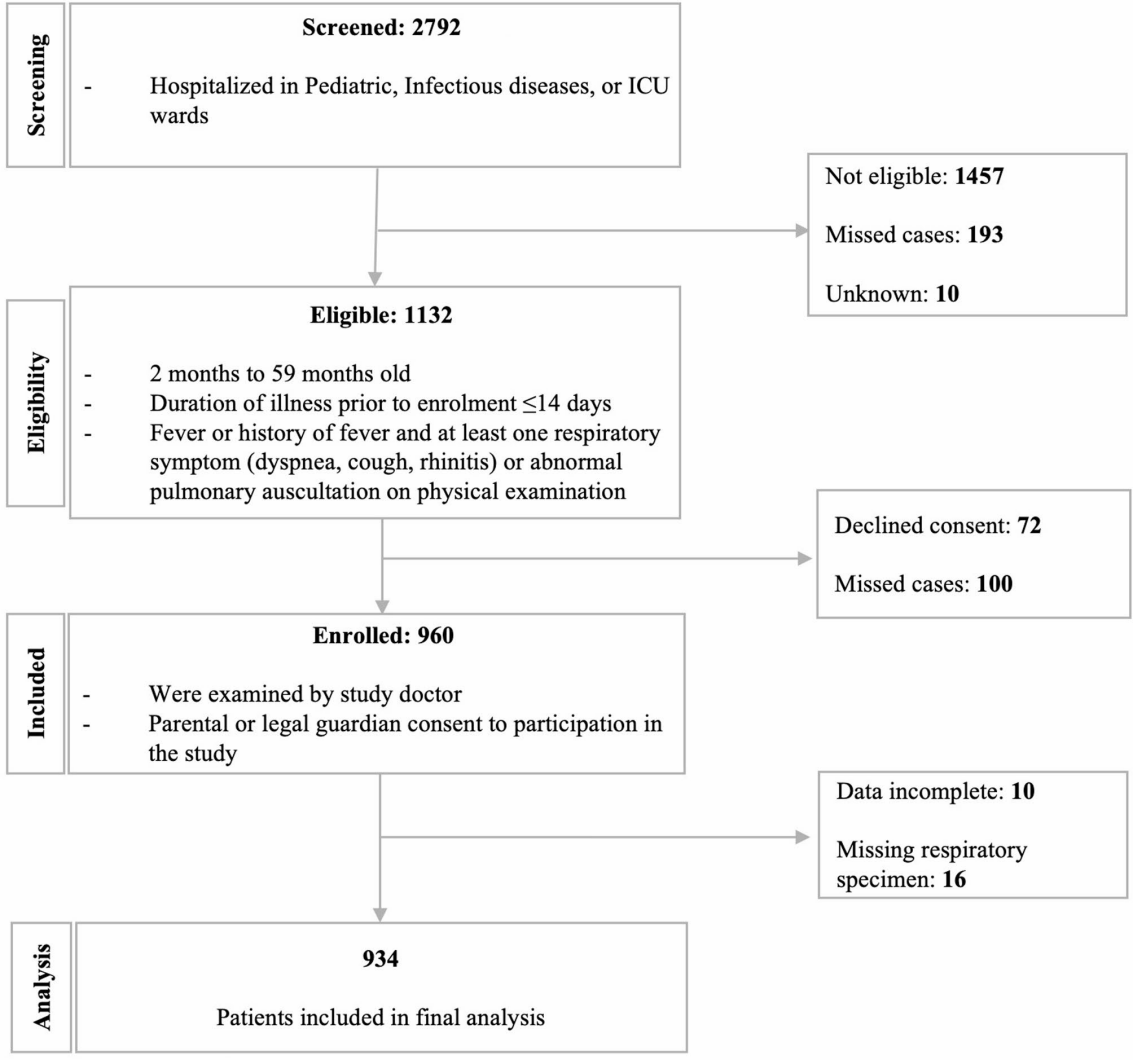
Flowchart of patient recruitment, December 2013 to December 2016.

A total of 2,792 children were hospitalized and 1,132 (40.4%) met the eligibility criteria for inclusion. Nine hundred and thirty four participants were included in the final analysis (Fig. 1). The median (IQR) age of participants was 14.7 (IQR 8.3–24.2) months, 73.9% (690/934) of participants were less than 2 years old (Table 1). All data necessary for pneumonia classification were available for 876/934 (93.8%) participants; 63.8% (559/876) of participants had pneumonia, 40.4% (354/876) of participants had severe pneumonia. Seventeen participants (2.0%) died.

**Table 1:** Demographic and clinical characteristics of study participants included in the study, by age groups.

Characteristics	All ARI (*n* = 934)
Demographics	
Age (month), median (IQR)	14.7 (8.3–24.2)
2 m to < 6 month	152 (16.3%)
6 m to < 1 year	220 (23.6%)
1 to < 2 year	318 (34.1%)
2 to < 5year	244 (26.1%)
Female, n (%)	404 (43.3)
Male, n (%)	530 (56.7)
Ethnicity, n (%)	
Lao loum	845 (90.5)
Hmong	77 (8.2)
Khmu	5 (0.5)
Other	7 (0.8)
Kindergarten attendance* ^*n*=911^, n (%)	196 (21.5)
Mother’s education ^*n*=921^, n (%)	
Primary school	109 (11.8)
Junior high school	183 (19.6)
High school	376 (40.6)
University	196 (21.3)
Illiterate	59 (6.4)
Mother education lower than university ^*n*=921^, n (%)	725 (78.7)
Ward, n (%)	
Paediatric general	679 (72.7)
IDP	156 (16.7)
ICUP	99 (10.6)
Wet season, n (%)	558 (59.7)
History	
Birth weight^*n*=853^ (kg), median (IQR)	3 (2.7–3.4)
Low birth weight ^*n*=853^, n (%)	103 (12.1)
Underweight^*n*=908^, n (%)	187 (20.6)
Patient was breast-fed^*n*=902^, n (%)	596 (66.1)
Born by Caesarean^*n*=933^, n (%)	129 (13.8)
Hot bed practice**^n=578^, n (%)	113 (19.6)
PCV 13 vaccination^*n*=847^, n (%)	401 (47.3)
Respiratory signs and symptoms	
Cough, n (%)	914 (97.9)
Runny nose, n (%)	838 (89.7)
Difficulty breathing ^*n*=924^, n (%)	625 (67.6)
Abnormal pulmonary auscultation ^*n*=915^, n (%)	637 (69.6)
Chest indrawing ^*n*=918^, n (%)	469 (51.1)
Respiratory rate (breaths/min) ^*n*=881^, median (IQR)	40 (30–53)
Fast breathing^& *n*=872^, n (%)	396 (45.4)
Respiratory distress ^*n*=912^, n (%)	162 (17.8)
Wheeze^£ *n*=913^, n (%)	284 (31.1)
Nasal flaring ^*n*=910^, n (%)	178 (19.6)
Stridor ^*n*=908^, n (%)	41 (4.4)
Cyanosis^ ^*n*=921^, n (%)	78 (8.5)
Oxygen saturation in room air (%) ^*n*=825^, median (IQR)	97.0 (94.0–98.0)
Oxygen saturation < 90% in room air ^*n*=825^, n (%)	102 (12.4)
Outcomes	
Pneumonia^● *n*=876^, n (%)	559 (63.8)
Severe pneumonia^● *n*=876^, n (%)	354 (40.4)
ICU admission required ^*n*=931^, n (%)	123 (13.4)
Supplementary oxygen used ^*n*=928^, n (%)	165 (17.8)
Mechanical Ventilation ^*n*=931^, n (%)	21 (2.3)
CPAP ^*n*=931^, n (%)	10 (1.1)
Total deaths ^*n*=854^, n (%)	17 (2.0)

*Kindergarten attendance: for children < 6 year old, if they attend kindergarten or day care.

^&^Fast breathing, for 2 to 11 months old: ≥ 50 breaths/min, 1 to 5 years old : ≥ 40 breaths/min (WHO pocket book of hospital care for children, 2013).

^**^ Hot bed practice: mother of the patient who practiced hot bed during few weeks after delivery.

^^^Cyanosis: central or peripheric.

^£^wheeze = history of wheeze or on admission.

^●^Pneumonia was defined as children with cough or difficulty breathing and fast breathing (aged 2–11 months: ≥50 breaths/minute, aged 1–4 years: ≥40 breaths/minute) or chest indrawing. Severe pneumonia was defined as children with cough or difficulty breathing who had at least one of the following criteria: oxygen saturation.

### Microorganism detection

Over the three year study period, RSV was the most frequent virus detected in 29.9% (279/934), whereas influenza viruses were detected in 9.2% (86/934) of participants. *H. influenzae* and *S. pneumoniae* were detected in 52.2% (497/934), and 37.7% (352/934) of participants, respectively (Table S1).

**Fig. 2:**
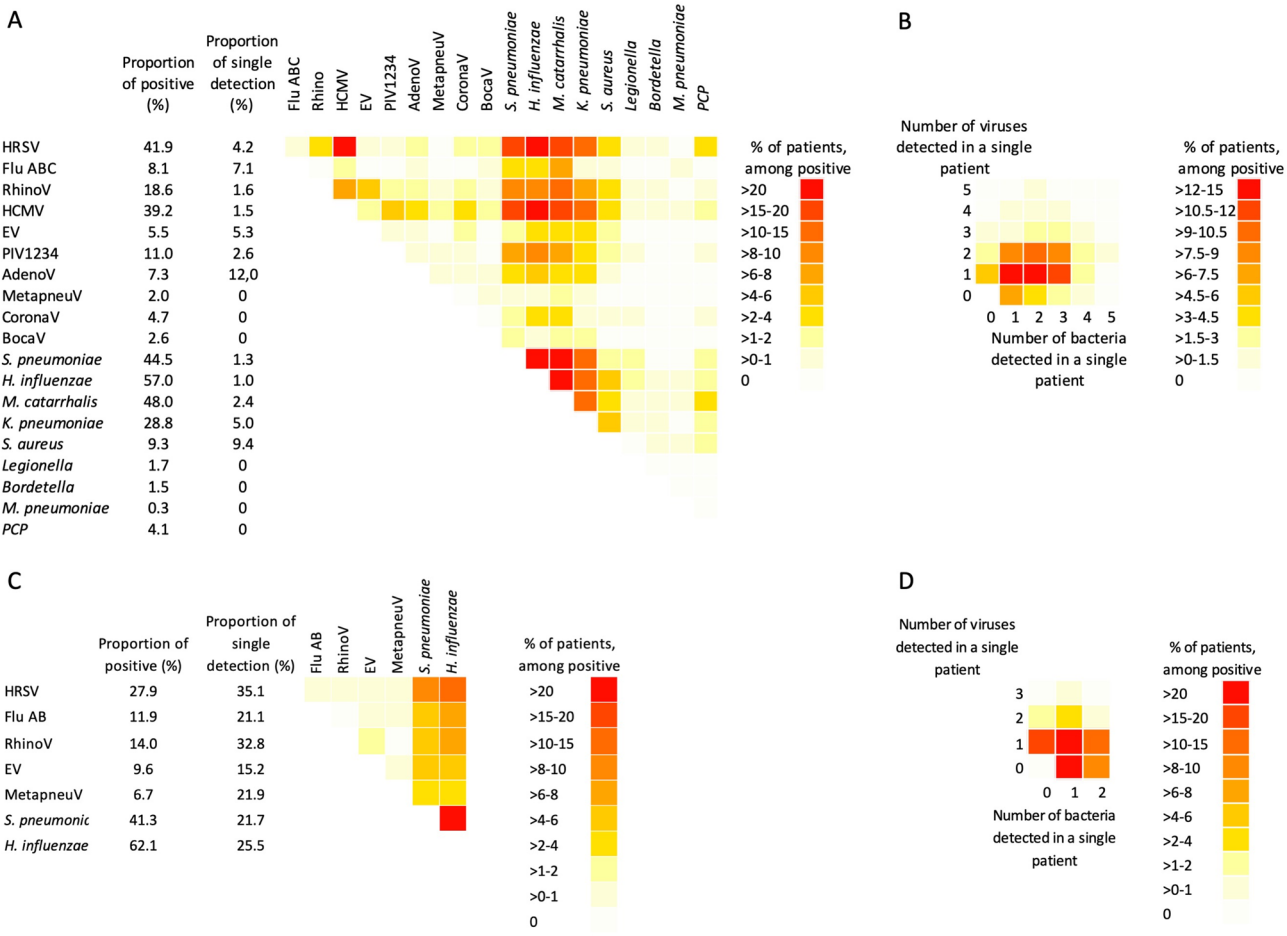
Co-detection of different microorganisms by real-time PCR in the same respiratory sample. (**A**) Co-detection for the 33 microorganisms tested for 349 participants (December 2013 to December 2014) using FTD33 kit. (**B**) Number of different viruses and bacteria detected in a single patient for the 349 patients tested using the FTD33 kit. (**C**) Co-detection for the eight microorganisms tested for 585 patients from January 2015 to December 2016. (**D**) Number of different viruses and bacteria detected in a single patient for the 585 patients tested for eight microorganisms.

From December 2013 to December 2014, among the 349 participants tested using the FTD33 kit (full microorganism detection described in separate manuscript, Bounvilay et al. in prep), co-detection (2 or more microorganisms detected in the same participant) was observed for 307 (88%) participants (Fig. 2).

From January 2015 to December 2016, among the 585 participants tested for 8 microorganisms, co-detection was observed for 265 (45.3%) participants (Fig. 2).

### Association between viral infection and increased pneumococcal density

In the 352 children with pneumococcal carriage (*n* = 110 with RSV detected), there was no association between RSV detection and *S. pneumoniae* density (adjusted RR 1.04, 95% CI: 0.45–2.39, supplemental Table S2 and S3). In contrast, *S. pneumoniae* density was higher by a factor of 2.41 in influenza virus positive participants when compared with influenza virus negative participants (95% CI: 1.34–4.36, *p* < 0.001) and this was confirmed when adjusted for potential confounders (adjusted RR 4.52, 95% CI 1.73 to 11.8, *p* < 0.001, supplemental Table S4).

### Association between co-detection and severe pneumonia

*S. pneumoniae*/*H. influenzae*, RSV/*S. pneumoniae* and RSV/*H. influenzae* co-detections were the most common microorganism combinations, found in 24.0% (224/934), 11.8% (110/934) and 16.1% (150/934) of included ARI patients, respectively. *S. pneumoniae*/*H. influenzae* co-detection was not associated with severe pneumonia (aOR 1.00, 95%CI 0.65 to 1.54, *p* = 0.994, Supplemental Table S5). Influenza virus/*S. pneumoniae* co-detection was observed in only 3.5% (33/934) of included ARI patients, therefore association with severe pneumonia was not analyzed.

RSV/*H. influenzae* co-detection was positively associated with severe pneumonia in the univariate model (OR 2.09, 95%CI 1.46 to 3.00, *p* < 0.001), but this association did not persist in the multivariable model (aOR 1.37, 95%CI 0.73 to 2.58, *p* = 0.322) (Supplemental Table S6).

Similarly, RSV/*S. pneumoniae* co-detection was positively associated with severe pneumonia (OR 1.86, 95%CI 1.22 to 2.81, *p* = 0.003) but after adjusting for confounders the risk of severe pneumonia was the same in children with or without co-detection of RSV/*S. pneumoniae* (aOR 0.72, 95% CI 0.38 to 1.6, *p* = 0.309) (Supplemental Table S7).

### In RSV positive patients, unadjusted association between Pneumococcal density and severe pneumonia

In RSV positive participants (*n* = 102), there was no difference in *S. pneumoniae* density (median (IQR) genome equivalent (GE)/mL) in participants with severe pneumonia (5.6 × 10^5^ (1.1 × 10^5^ to 2.0 × 10^6^), *n* = 55) and participants without severe pneumonia (1.4 × 10^6^ (1.6 × 10^5^ to 3.0 × 10^6^) *n* = 47, *p* = 0.096).

## Discussion

In our study, we found higher pneumococcal colonization density in influenza virus positive participants in comparison to participants who were influenza virus negative. This is in accordance with findings in animal models^36–39^ and epidemiological studies^7,40^. For example, a clinical study in Vietnam of 106 children with confirmed pneumonia found that, in children with viral infection (influenza virus, rhinovirus or RSV), pneumococcal density in URT was 15 fold higher than in children with no viral infection, similar to what we observed here for influenza virus infection. Influenza A virus and RSV were associated with an increased pneumococcal density in the URT^40^. In a study of 969 patients (all ages) recruited in 2010 in South Africa as part of a study of severe ARI, Wolter *et al*. observed that patients with viral coinfection (influenza virus, adenovirus, parainfluenza virus, rhinovirus) had higher pneumococcal colonization densities than patients with no viral coinfection^7^. In another study in Peru during 2009–2011, among 450 ARI samples collected from children in the community, there was no difference in pneumococcal colonization density between the 299 virus positive samples and the 136 virus negative samples. However, higher pneumococcal density was observed in samples in which rhinovirus was the only virus detected in comparison to samples with no virus detected^4^.

In contrast, our study found that pneumococcal density was not higher in children with RSV. However, we may have had too few cases to detect a difference. In comparison with influenza virus, less is understood about the causal link between RSV infection and pneumococcal density in the URT. In the study from Mitsi *et al*. RSV infection led to a transient increase in pneumococcal density^41^. Their research involved healthy adult participants who were experimentally challenged with live pneumococci of serotype 6B to assess colonization. In contrast, our study included hospitalized children with ARI, analyzing URT samples via PCR, which did not allow differentiation between pneumococcal carriage and pathogenic infection. Therefore, in our study, elevated pneumococcal density may be influenced by additional factors beyond RSV infection, potentially reducing the observed impact of RSV on pneumococcal load. Our results contrast with an infant mouse model that showed that infection with an RSV murine analogue increased pneumococcal density in the nasopharynx^42^. Although increase in density was observed for the two pneumoccocal strains tested, timing of the sampling was important. The increase in density was significant only in samples taken between 5 and 8 days post viral infection for the EF3030 strain. At 11 days post viral infection, pneumococcal density was significantly higher only for the BCH19 strain. This would suggest that the increase in pneumoccal density induced by RSV infection would be limited in the early stage of the viral infection, and the magnitude would vary according to the pneumococcal strains colonizing the nasopharynx. This might explain why we did not observe a significant increase in pneumococcal density in acute patients infected by RSV recruited in our study, as sampling was only conducted at a single time point. Data on pneumococcal serotype colonizing the nasopharynx were not available, and serotype might play a role in RSV-pneumococcal interactions, as indicated from a study of children with CAAP in Israel^43^.

RSV is known to play an important role in the course of pneumococcal pneumonia in young children. However, in our study, we found that a higher pneumococcal density was not a predictor of severe pneumonia in RSV positive patients. These results contrast with some other studies which have found a positive association between RSV and higher pneumococcal density, and a positive association between higher pneumococcal density and more severe disease. Esposito *et al*. (2013) found a positive association between pneumococcal density and alveolar involvement in RSV positive children with community acquired pneumonia^44^. However, in the study of Vissers *et al*. conducted among 105 children hospitalized with RSV infection, severely ill patients had lower pneumococcal densities than other non-severely ill patients^45^. Studies conducted in France and in Israel showed that COVID-19 pandemic measures led to a substantial decrease in RSV, influenza virus and metapneumovirus circulation^10–12^. This was associated with a decrease in pneumococcal disease incidence. However, nasopharyngeal pneumococcal carriage was not affected by the public health measures, with the mean pneumococcal density within the range of what was observed before the pandemic^10^. In contrast, in Vietnam, a decrease in pneumococcal density was observed in two year old children after the implementation of non-pharmaceutical interventions in 2020^46^, likely coinciding with a decline in the circulation of seasonal respiratory viruses, as reported in other countries^47–50^. Despite contrasting findings in relation to respiratory viruses and pneumococcal density, the findings of all studies support a link between viral infection and pneumococcal disease that may not be universally driven by increased pneumococcal density in the URT.

We did not find any associations between *S. pneumoniae* and *H. influenzae* co-detection and severe pneumonia. Our findings are in contrast with other studies, although these had different designs. In a case-control study, Ngocho and colleague observed increased odds for being colonized with *S. pneumoniae* in children co-colonized with *M. catarrhalis* and *H. influenzae*^51^. They also observed that co-detection of *S. pneumoniae* and *H. influenzae* was associated with higher odds for developing pneumonia, supporting the suggestion that there could be a synergy between *S. pneumoniae* and *H. influenzae*. We cannot exclude that children who have other risk factors for pneumonia (malnutrition, poverty, etc.) are also at higher risk for pneumococcal and *H. influenzae* carriage. In a study of 550 hospitalized children with LRT infections in Vietnam, including 274 with confirmed pneumonia and 350 healthy controls, Vu *et al*. found that *S. pneumoniae/H. influenzae* codetection was associated with pneumonia^40^. Diaz-Diaz *et al*. found in children less than 2 years old with RSV infection, that *S. pneumoniae/H. influenzae* codetection was associated with more severe disease (greater odds of hospitalization, needs for supplemental oxygen or longer hospitalization)^52^.

Although we observed an association between RSV/*S. pneumoniae* co-detection and severe pneumonia and between RSV/*H. influenzae* co-detection and severe pneumonia in univariate analysis, those findings were not confirmed by adjusted results. Severe pneumonia is multifactorial, and other factors such as host immunity, genetic predispositions, or environmental factors may play a more dominant role in determining disease severity. Secondary bacterial superinfections following viral infections are well documented. Several pathways have been proposed to explain the underlying mechanisms. Respiratory viruses are likely to alter the microbial flora of the URT. Following alteration of the microbial community in the URT, respiratory viruses may promote colonization of the LRT through mechanisms impairing bacterial clearance. Viral infection might also enhance growth of *S. pneumonia*e, leading to bacterial superinfection^6,15,16^. Growing evidence suggests that bidirectional mechanisms may be involved^53^. Viral replication in the URT may be boosted by the presence of *S. pneumoniae*^14^. However, some studies have shown lower viral load in the presence pneumococcal carriage^42^. A positive association between the pneumococcus and the severity of RSV disease has been well documented^17^. However, several studies did not demonstrated an association between RSV/*S. pneumonia*e co-detection and disease severity. Patient inclusion criteria and criteria for severity are often different from one study to another, making it difficult to evaluate the impact of RSV/*S. pneumoniae* co-detection on clinical severity^17^. In the PERCH study, a higher proportion of bacteria (*S. pneumoniae* and *H. influenzae*) in very severe pneumonia compared to severe pneumonia was observed, but mixed bacteria/virus detection was high in both cases (83.5%) and controls (75.8%). However, the analysis model used in the PERCH study did not take into account multiple pathogens as a potential cause of infection. In a study of 307 children < 5 years old hospitalized with severe pneumonia, between 2014 and 2016 in Malaysia, co-infection was not found to be associated with criteria associated with more severe disease, such as length of hospitalization or supplemental respiratory support^54^.

Our study has some limitations: the FTD33 kit was used only for the first year of the study, no control group was included in the study, and no samples were collected from the LRT. In addition, only 59 RSV positive patients with severe pneumonia had received PCV, and therefore our data set did not allow us to explore whether PCV was protective against RSV related severe pneumonia. Only 33 patients presented with Influenza virus/*S. pneumoniae* co-detection, therefore our data did not permit robust exploration of the impact of Influenza virus/*S. pneumoniae* co-detection on disease severity.

This study was conducted before the COVID-19 pandemic. More research and ARI surveillance systems will be vital for detecting new endemic and epidemic microorganisms. COVID-19 is likely to become an endemic pathogen that will need to be included in diagnostic and treatment algorithms. How COVID-19 and the human responses (such as physical distancing and reduced human clustering) change the epidemiology of ARI globally and in Laos will need to be investigated.

## Conclusions

No evidence of an association between RSV and pneumococcal detection and density was observed in our study, highlighting the complexity of the interaction of those pathogens in the course of pneumonia development rather than a simple synergistic action. However, RSV epidemiology is likely to evolve in Laos in COVID post-pandemic context. Further studies are needed to evaluate of the impact of PCV implementation on RSV hospitalization. Since 2024, RSV vaccine for pregnant women and monoclonal antibodies are available to protect infants and young children against severe RSV infection. Studies are needed to estimate their impact on pediatric acute lower respiratory tract infection, hospitalizations, including their role in preventing pneumococcal pneumonia, in LMICs.

## Electronic supplementary material

Below is the link to the electronic supplementary material.

Supplementary Material 1

## Data Availability

The datasets used and/or analysed during the current study are available from the corresponding author on reasonable request.
