# Supplementary material for: Pneumococcal density and respiratory co-detection in severe pediatric pneumonia in Laos
Source: Sci Rep. 2025 May 21;15:17708. doi: 10.1038/s41598-025-01659-y (PMC12095513; doi:10.1038/s41598-025-01659-y)
Supplement: Supplementary file 1 — Supplementary Material 1 [file 41598_2025_1659_MOESM1_ESM.docx]

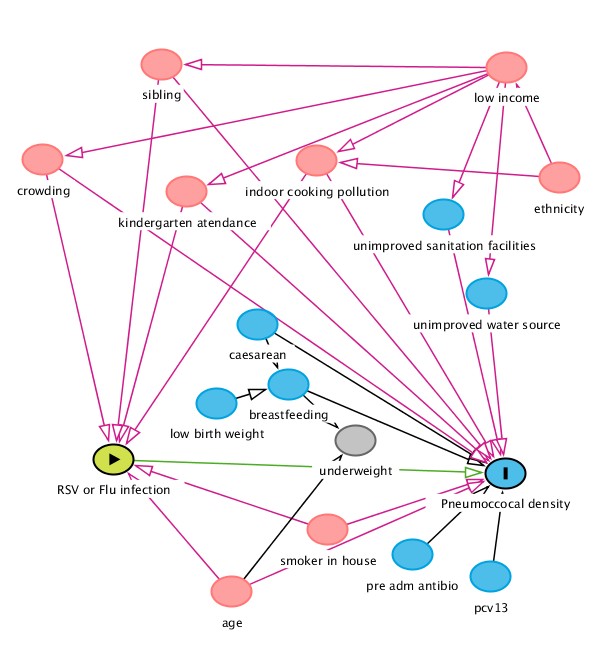


**Figure S1**: Directed acyclic graphs for the association of viral detection with *S. pneumoniae* density. Directed acyclic graphs established for the models used to investigate the association of viral detection (RSV or influenza virus) with *S. pneumoniae* density as outcome.


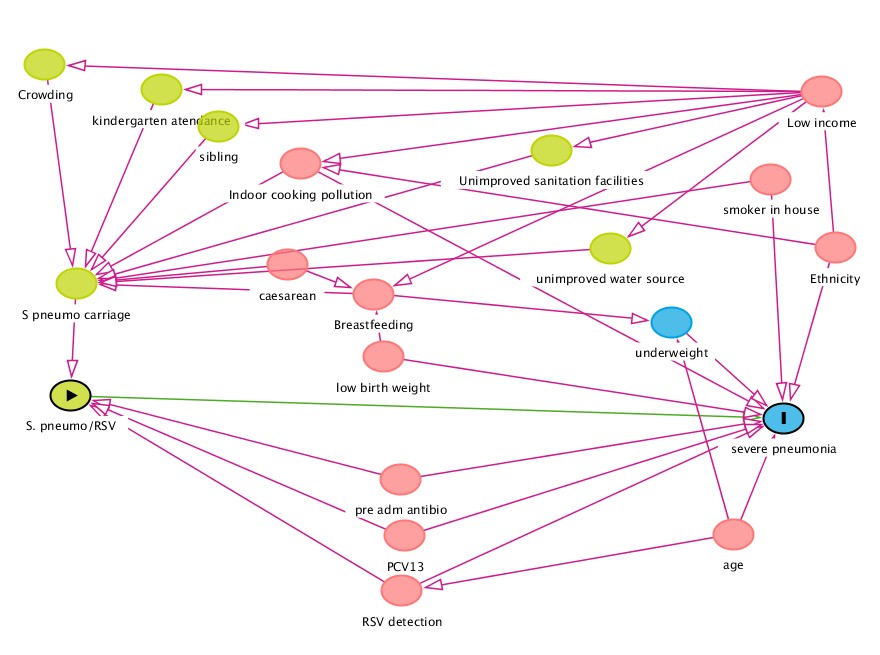


**Figure S2**: Directed acyclic graphs established for associations of co-detection with severe pneumonia. Directed acyclic graphs established for the models used to quantify associations between co-detection (RSV/*S. pneumoniae*, or RSV/*H. influenzae*, or *H. influenzae*/ *S. pneumoniae*) and severe pneumonia.

**Table S1: Distribution of the micro-organisms detected per year by RT-qPCR in hospitalized children included in the study.**

| **Pathogens** | **Years** | | | | **Total**  **n=934** |
| --- | --- | --- | --- | --- | --- |
|  | **2013^#^**  **n=6** | **2014^#^**  **n=344** | **2015**  **n=309** | **2016**  **n=275** |  |
| Viruses |  |  |  |  |  |
| HRSVA/B | 4 (66.7) | 141 (41.0) | 35 (11.3) | 99 (36.0) | 279 (29.9) |
| HRV | 1 (16.7) | 63 (18.3) | 46 (14.9) | 20 (7.3) | 130 (13.9) |
| EV** | 0 | 19 (5.5) | 27 (8.7) | 19 (6.9) | 65 (7.0) |
| Influenza virus | 0 | 29 (8.4) | 25 (8.1) | 32 (11.5) | 86 (9.2) |
| Influenza A virus | 0 | 12 (3.5) | 21 (6.8) | 16 (5.8) | 49 (5.3) |
| Influenza B virus | 0 | 15 (4.4) | 5 (1.6) | 16 (5.8) | 36 (3.9) |
| Influenza C virus | 0 | 2 (0.6) ^♦^ |  |  | 2 (0.6) ^♦^ |
| HMPVA/B | 0 | 7 (2.0) | 28 (9.2) | 4 (1.5) | 39 (4.2) |
| HCMV | 2 (33.3) | 132 (38.5)^♦^ |  |  |  |
| Parainfluenza | 0 | 37 (10.8) ^♦^ |  |  |  |
| Parainfluenza 1 | 0 | 7 (2.0) ^♦^ |  |  |  |
| Parainfluenza 2 | 0 | 2 (0.6) ^♦^ |  |  |  |
| Parainfluenza 3 | 0 | 23 (6.7) ^♦^ |  |  |  |
| Parainfluenza 4 | 0 | 5 (1.5) ^♦^ |  |  |  |
| Human adenovirus | 0 | 25 (7.3) ^♦^ |  |  |  |
| Coronavirus | 1 (16.7) | 15 (4.4) ^♦^ |  |  |  |
| Cor OC43 | 1 (16.7) | 8 (2.3) ^♦^ |  |  |  |
| Cor NL63 | 0 | 6 (1.8) ^♦^ |  |  |  |
| Cor HKU1 | 0 | 1 (0.3) ^♦^ |  |  |  |
| Cor 229E | 0 | 0^♦^ |  |  |  |
| Bocavirus | 0 | 8 (2.3) ^♦^ |  |  |  |
|  |  |  |  |  |  |
| Bacteria |  |  |  |  |  |
| *S. pneumoniae* | 2 (33.3) | 152 (44.2) | 112 (36.3) | 86 (31.4) | 352 (37.7) |
| *H. influenzae* | 2 (33.3) | 197 (57.3)*** | 162 (52.4) | 136 (49.5) | 497 (53.2) |
| *M. catarrhalis* | 4 (66.7) | 160 (46.7) ^♦^ |  |  |  |
| *K. pneumoniae* | 1 (16.7) | 96 (28.0) ^♦^ |  |  |  |
| *S. aureus* | 0 | 32 (9.3) ^♦^ |  |  |  |
| *Legionella* | 0 | 6 (1.8) ^♦^ |  |  |  |
| *C. pneumoniae* | 0 | 0^♦^ |  |  |  |
| *Bordetella spp* | 0 | 5 (1.5) ^♦^ |  |  |  |
| *Salmonella spp* | 0 | 0^♦^ |  |  |  |
| *M. pneumoniae* | 0 | 1 (0.3) ^♦^ |  |  |  |
| Fungi |  |  |  |  |  |
| *P. jirovecii* | 1 (16.7) | 13 (3.8) ^♦^ |  |  |  |

^#^From December 2013 to December 2014, 349 patients were tested using FTD33 kit, the last patient recruited in December 2014 was tested for 8 pathogens using singleplex q(RT-)PCRs. Mico-organism detection was defined as Cq<35 for the corresponding RT-qPCR.

**^♦^** tested for 343 patients

** including Parechovirus for the 349 tested using FTD (6 patients in 2013 and 343 in 2014).

*** including 14 *H. influenzae* type B

**Table S2 : Univariate analysis to identify factors associated with *S. pneumoniae* load (n=352).** Factors included in multivariate model in bold.

|  | Rate ratio | p-value | Lower 95% CI | Upper 95% CI |
| --- | --- | --- | --- | --- |
| RSV detection* | 0.82 | 0.29 | 0.56 | 1.19 |
| **Age (years)** | **0.19** | **0.04** | **1.00** | **1.41** |
| **Male** | **1.46** | **0.03** | **1.03** | **2.07** |
| Underweight | 0.93 | 0.75 | 0.60 | 1.45 |
| PCV13 vaccine received | 0.89 | 0.55 | 0.62 | 1.29 |
| Breastfeeding | 0.98 | 0.91 | 0.68 | 1.41 |
| Caesarean delivery | 1.14 | 0.61 | 0.69 | 1.88 |
| Low birth weight | 1.30 | 0.38 | 0.72 | 2.35 |
| **Hot bed practice (for < 6mo)**** | **2.78** | **0.09** | **0.84** | **9.21** |
| **Kindergarten attendance** | **1.78** | **0.01** | **1.18** | **2.68** |
| **Siblings (other children<5y old)** | **21.8** | **0.01** | **2.18** | **217.9** |
| More than 2 people/sleeping room | 0.85 | 0.46 | 0.54 | 1.31 |
| **Low family income** | **0.57** | **<0.01** | **0.40** | **0.81** |
| **Source of indoor cooking fuel***** | **1.86** | **0.02** | **1.12** | **3.09** |
| **Smoker in the house** | **0.79** | **0.19** | **0.55** | **1.12** |
| Unimproved water source | 1.11 | 0.74 | 0.59 | 2.10 |
| **Unimproved sanitation facilities** | **0.45** | **0.01** | **0.25** | **0.83** |
| **AB use before admission** | **0.74** | **0.10** | **0.52** | **1.05** |
| **Ethnicity** |  |  |  |  |
| Lao loum | Ref group |  |  |  |
| Hmong | 1.09 | 0.78 | 0.58 | 2.05 |
| Other | **0.04** | **<0.01** | **0.01** | **0.19** |

* Univariate result showed no association between RSV detection and *S. pneumoniae* load.

** n=31

*** n=202

**Table S3: Multivariate analysis of the association of RSV detection and pneumococcal density.**

|  | Including source of indoor cooking fuel in the model, n=173 | | | | Excluding source of indoor cooking fuel from the model, n=294 | | | |
| --- | --- | --- | --- | --- | --- | --- | --- | --- |
|  | Rate ratio | p-value | Lower 95% CI | Upper 95% CI | Rate ratio | p-value | Lower 95% CI | Upper 95% CI |
| **RSV** | 1.04 | 0.93 | 0.45 | 2.39 | 1.15 | 0.55 | 0.73 | 1.83 |
| Age (years) | 0.90 | 0.48 | 0.66 | 1.21 | 1.07 | 0.52 | 0.86 | 1.34 |
| Male | 1.61 | 0.11 | 0.90 | 2.88 | 1.18 | 0.41 | 0.79 | 1.76 |
| Kindergarten attendance | 1.40 | 0.39 | 0.65 | 3.01 | 1.39 | 0.26 | 0.79 | 2.46 |
| Siblings (other children<5y old) | 11401 | <0.01 | 267 | 486091 | 30.7 | <0.01 | 3.13 | 300.3 |
| Low family income | 0.98 | 0.95 | 0.54 | 1.79 | 0.63 | 0.03 | 0.42 | 0.96 |
| Source of indoor cooking fuel | 1.88 | 0.06 | 0.98 | 3.61 | - | - | - | - |
| Smoker in the house | 0.59 | 0.06 | 0.34 | 1.03 | 0.61 | 0.02 | 0.41 | 0.91 |
| Unimproved sanitation facilities | 2.54 | 0.35 | 0.36 | 17.8 | 0.76 | 0.45 | 0.37 | 1.56 |
| AB use before admission | 0.50 | 0.01 | 0.29 | 0.87 | 0.60 | 0.01 | 0.40 | 0.90 |
| Ethnicity Lao loum | Ref group |  |  |  | Ref group |  |  |  |
| Hmong | 1.42 | 0.46 | 0.56 | 3.59 | 1.21 | 0.61 | 0.58 | 2.52 |
| Other | 0.06 | <0.01 | 0.01 | 0.33 | 0.04 | <0.01 | 0.01 | 0.20 |
| More than 2 people/sleeping room | 0.85 | 0.68 | 0.41 | 1.79 | 0.96 | 0.89 | 0.57 | 1.63 |

The association between pneumococcal density and RSV was quantified using negative binomial regression with pneumococcal carriage density as the outcome. Only pneumococcal carriers were included. The multivariable model included variables found to be significant at the univariable level (p<0.2) and those identified using a directed acyclic graph (Supplemental Figure S1).

**Table S4: Multivariate analysis of the association of influenza virus detection and pneumococcal density.**

|  | Including source of indoor cooking fuel in the model, n=173 | | | | Excluding source of indoor cooking fuel from the model, n=294 | | | |
| --- | --- | --- | --- | --- | --- | --- | --- | --- |
|  | Rate ratio | p-value | Lower 95% CI | Upper 95% CI | Rate ratio | p-value | Lower 95% CI | Upper 95% CI |
| **Flu** | 4.52 | <0.01 | 1.73 | 11.8 | 2.90 | <0.01 | 1.48 | 5.70 |
| Age (years) | 0.79 | 0.11 | 0.60 | 1.05 | 0.97 | 0.81 | 0.77 | 1.22 |
| Male | 1.50 | 0.13 | 0.88 | 2.55 | 1.08 | 0.69 | 0.73 | 1.60 |
| Kindergarten attendance | 1.25 | 0.53 | 0.62 | 2.54 | 1.37 | 0.26 | 0.79 | 2.38 |
| Siblings (other children<5y old) | 1559 | <0.01 | 33.6 | 72424 | 24.1 | 0.01 | 2.53 | 229.9 |
| Low family income | 1.18 | 0.57 | 0.66 | 2.13 | 0.83 | 0.40 | 0.54 | 1.28 |
| Source of indoor cooking fuel | 1.24 | 0.52 | 0.65 | 2.36 | - | - | - | - |
| Smoker in the house | 0.56 | 0.03 | 0.34 | 0.94 | 0.53 | <0.01 | 0.35 | 0.79 |
| Unimproved sanitation facilities | 0.54 | 0.57 | 0.07 | 4.39 | 0.56 | 0.12 | 0.27 | 1.16 |
| AB use before admission | 0.42 | <0.01 | 0.25 | 0.71 | 0.61 | 0.02 | 0.41 | 0.91 |
| Ethnicity Lao loum | Ref group |  |  |  | Ref group |  |  |  |
| Hmong | 1.41 | 0.46 | 0.57 | 3.49 | 1.13 | 0.74 | 0.55 | 2.33 |
| Other | 0.06 | <0.01 | 0.01 | 0.31 | 0.03 | <0.01 | 0.01 | 0.17 |
| More than 2 people/sleeping room | 0.62 | 0.15 | 0.33 | 1.18 | 0.75 | 0.25 | 0.46 | 1.22 |

The association between pneumococcal density and influenza virus was quantified using negative binomial regression with pneumococcal carriage density as the outcome. Only pneumococcal carriers were included. The multivariable model included variables found to be significant at the univariable level (p<0.2) and those identified using a directed acyclic graph (supplemental Figure S1).

**Table S5: Univariate and Multivariate analysis of the association of *S. pneumoniae*/*H. influenzae* detection with WHO severe pneumonia.**

| **Univariate analysis** | | | | | | | | | |
| --- | --- | --- | --- | --- | --- | --- | --- | --- | --- |
|  | All ARI patients  N=934 | ARI patient with WHO severe pneumonia*  N=354 | | | ARI patient without severe pneumonia  N=521 | | P value | | Odds Ratio (95%CI) |
| *S. pneumoniae/H. influenzae* co-detection, n (%) | 224 (24.0%) | 85 (24.0%) | | | 126 (24.2%) | | 0.953 | | 0.99  (0.72 – 1.36) |
| **Multivariate analysis** | | | | | | | | | |
|  | | | Excluding source of indoor cooking fuel from the model, n=612* | | | | | | |
| WHO severe pneumonia | | | Odd Ratio | p-value | | Lower 95% CI | | Upper 95% CI | |
| *S. pneumoniae*/*H. influenzae* co-detection | | | **1.002** | **0.994** | | **0.653** | | **1.537** | |
| Age (years) | | | 0.685 | 0.001 | | 0.550 | | 0.853 | |
| Underweight | | | 1.480 | 0.081 | | 0.954 | | 2.296 | |
| Breastfeeding | | | 1.864 | 0.002 | | 1.258 | | 2.762 | |
| Caesarean delivery | | | 0.627 | 0.072 | | 0.377 | | 1.043 | |
| Low birth weight | | | 2.745 | <0.001 | | 1.601 | | 4.707 | |
| Kindergarten attendance | | | 1.019 | 0.945 | | 0.601 | | 1.726 | |
| Crowding | | | 0.768 | 0.232 | | 0.499 | | 1.184 | |
| Source of indoor cooking fuel | | | 1.196 | 0.341 | | 0.828 | | 1.729 | |
| Smoker in the house | | | 2.695 | 0.002 | | 1.437 | | 5.054 | |
| Unimproved water source | | | 1.068 | 0.858 | | 0.518 | | 2.204 | |
| Unimproved sanitation facilities | | | 1.181 | 0.366 | | 0.823 | | 1.695 | |
| AB use before admission | | |  |  | |  | |  | |
| Ethnicity Lao loum | | | 1.838 | 0.085 | | 0.920 | | 3.671 | |
| Hmong | | | 1.664 | 0.525 | | 0.346 | | 8.004 | |
| Other | | | 1.885 | 0.001 | | 1.276 | | 2.785 | |
| RSV detection | | | 0.848 | 0.420 | | 0.568 | | 1.265 | |
| Low family income | | | 0.845 | 0.395 | | 0.574 | | 1.245 | |
| PCV13 vaccine received | | | 0.849 | 0.406 | | 0.577 | | 1.249 | |

Multivariable model included variables found to be significant at the univariable level (p<0.2) and those identified using a directed acyclic graph (supplemental Figure S2).

*Similar results were obtained when the analysis was performed by including indoor cooking fuel in the model (n=409), data not shown.

**Table S6: Univariate and multivariate analysis of the association of RSV/*H. influenzae* detection with WHO severe pneumonia.**

| **Univariate analysis** | | | | | | |
| --- | --- | --- | --- | --- | --- | --- |
|  | All ARI patients  N=934 | ARI patient with WHO severe pneumonia*  N=354 | | ARI patient without severe pneumonia  N=521 | P value | Odds Ratio (95%CI) |
| RSV/*H. influenzae* co-detection, n (%) | 150 (16.1%) | 80 (22.6%) | | 64 (12.3%) | <0.001 | 2.09  (1.46 - 3.00) |
| **Multivariate analysis** | | | | | | |
|  | | | Excluding source of indoor cooking fuel from the model, n=612* | | | |
| WHO severe pneumonia | | | Odd Ratio | p-value | Lower 95% CI | Upper 95% CI |
| RSV/*H. influenzae* co-detection | | | **1.374** | **0.322** | **0.733** | **2.576** |
| Age (years) | | | 0.681 | 0.001 | 0.546 | 0.850 |
| Underweight | | | 1.498 | 0.072 | 0.965 | 2.325 |
| Breastfeeding | | | 1.858 | 0.002 | 1.254 | 2.751 |
| Caesarean delivery | | | 0.627 | 0.072 | 0.377 | 1.043 |
| Low birth weight | | | 2.772 | <0.001 | 1.618 | 4.749 |
| Kindergarten attendance | | | 0.992 | 0.977 | 0.587 | 1.677 |
| Crowding | | | 0.767 | 0.229 | 0.497 | 1.182 |
| Source of indoor cooking fuel | | |  |  |  |  |
| Smoker in the house | | | 1.191 | 0.351 | 0.824 | 1.721 |
| Unimproved water source | | | 2.671 | 0.002 | 1.423 | 5.014 |
| Unimproved sanitation facilities | | | 1.048 | 0.900 | 0.507 | 2.166 |
| AB use before admission | | | 1.191 | 0.337 | 0.833 | 1.703 |
| Ethnicity Lao loum | | |  |  |  |  |
| Hmong | | | 1.849 | 0.082 | 0.926 | 3.692 |
| Other | | | 1.593 | 0.565 | 0.326 | 7.774 |
| RSV detection | | | 1.578 | 0.088 | 0.934 | 2.669 |
| Low family income | | | 0.842 | 0.399 | 0.565 | 1.255 |
| PCV13 vaccine received | | | 0.849 | 0.406 | 0.577 | 1.249 |

Multivariable model included variables found to be significant at the univariable level (p<0.2) and those identified using a directed acyclic graph (supplemental Figure S2).

*Similar results were obtained when the analysis was performed by including indoor cooking fuel in the model (n=409), data not shown.

**Table S7: Univariate and multivariate analysis of the association of RSV/S. pneumoniae detection with WHO severe pneumonia.**

| **Univariate analysis** | | | | | | | | |
| --- | --- | --- | --- | --- | --- | --- | --- | --- |
|  | All ARI patients  N=934 | ARI patient with WHO severe pneumonia*  N=354 | | ARI patient without severe pneumonia  N=521 | | P value | | Odds Ratio (95%CI) |
| RSV/*S. pneumoniae* co-detection, n (%) | 110 (11.8) | 55 (15.5%) | | 47 (9%) | | 0.003 | | 1.86  (1.22 - 2.81) |
| **Multivariate analysis** | | | | | | | | |
|  | | | Excluding source of indoor cooking fuel from the model, n=612* | | | | | |
| WHO severe pneumonia | | | Odds Ratio | p-value | Lower 95% CI | | Upper 95% CI | |
| RSV/*S. pneumoniae* co-detection | | | **0.716** | **0.309** | **0.376** | | **1.363** | |
| Age (years) | | | 0.684 | 0.001 | 0.549 | | 0.852 | |
| Underweight | | | 1.464 | 0.090 | 0.943 | | 2.272 | |
| Breastfeeding | | | 1.883 | 0.002 | 1.270 | | 2.792 | |
| Caesarean delivery | | | 0.630 | 0.075 | 0.378 | | 1.048 | |
| Low birth weight | | | 2.731 | <0.001 | 1.591 | | 4.688 | |
| Kindergarten attendance | | | 1.017 | 0.950 | 0.603 | | 1.714 | |
| Crowding | | | 0.755 | 0.203 | 0.489 | | 1.164 | |
| Source of indoor cooking fuel | | |  |  |  | |  | |
| Smoker in the house | | | 1.207 | 0.318 | 0.835 | | 1.744 | |
| Unimproved water source | | | 2.705 | 0.002 | 1.442 | | 5.076 | |
| Unimproved sanitation facilities | | | 1.050 | 0.895 | 0.509 | | 2.169 | |
| AB use before admission | | | 1.171 | 0.386 | 0.819 | | 1.674 | |
| Ethnicity Lao loum | | |  |  |  | |  | |
| Hmong | | | 1.878 | 0.075 | 0.939 | | 3.753 | |
| Other | | | 1.619 | 0.550 | 0.333 | | 7.861 | |
| RSV detection | | | 2.136 | 0.001 | 1.351 | | 3.376 | |
| Low family income | | | 0.868 | 0.491 | 0.581 | | 1.297 | |
| PCV13 vaccine received | | | 0.844 | 0.390 | 0.573 | | 1.242 | |

Multivariable model included variables found to be significant at the univariable level (p<0.2) and those identified using a directed acyclic graph (supplemental Figure S2).

*Similar results were obtained when the analysis was performed by including indoor cooking fuel in the model (n=409), data not shown.
